# Supplementary material for: Type 2 Diabetes Mellitus Coincident with Clinical and Subclinical Thyroid Dysfunctions Results in Dysregulation of Circulating Chemerin, Resistin and Visfatin
Source: Biomedicines. 2023 Jan 25;11(2):346. doi: 10.3390/biomedicines11020346 (PMC9952980; doi:10.3390/biomedicines11020346)
Supplement: Supplementary file 1 [file biomedicines-11-00346-s001.zip › biomedicines-1877625-supplementary.docx]

**Table S1.** Comparison of serum adipocytokine-concentrations, inflammatory-factor values and HOMA-IR in T2DM patients with thyroid dysfunctions. All patients were divided into two groups, based on BMI (BMI < 25 kg/m^2^, and BMI > 25 kg/m^2^), and * and ** indicate significant differences between the two groups at *p* < 0.05 and *p* < 0.01.

| **T2DM with Thyroid Dysfunctions** | | |
| --- | --- | --- |
| BMI > 25 kg/m^2^ | BMI < 25 kg/m^2^ | Variables |
| 29.51 ± 1.60 * | 23.78 ± 1.34 | BMI |
| 33.89 ± 6.7 ** | 18.21 ± 2.02 | Visfatin |
| 37.83 ± 4.32 * | 26.8 ± 8.8 | Resistin |
| 333.04 ± 62.81 * | 278.9 ± 54.25 | Chemerin |
| 6.47 ± 1.43 * | 5.01 ± 0.65 | CRP |
| 59.90 ± 15.72 ** | 41.19 ± 11.31 | IL-6 |
| 19.51 ± 3.59 * | 12.91 ± 2.72 | IL1-β |
| 6.79 ± 0.78 * | 5.52 ± 0.61 | HOMA-IR |

CRP: C- Reactive protein; HOMA-IR: homeostatic-model assessment for insulin resistance; BMI: body mass index.
